# Spectral, thermal studies and biological activity of pyrazinamide complexes

**DOI:** 10.1016/j.heliyon.2019.e02912

**Published:** 2019-11-29

**Authors:** Alaa E. Ali, Gehan S. Elasala, Essam A. Mohamed, Sherif A. Kolkaila

**Affiliations:** a Chemistry Department, Faculty of Science, Damanhour University, Damanhour, Egypt; b Chemistry Department, Faculty of Science and Arts, Shaqra University, Sajir, Saudi Arabia

**Keywords:** Analytical chemistry, Inorganic chemistry, Decomposition mechanisms, Thermal analysis, Pyrazinamide, Complexes, Biological activity, Structural chemistry

## Abstract

Synthesis and spectrothermal characterization of new fabricated pyrazinamide complexes with metal [Cr(III), Mn(II), Fe(III), Co(II), Ni(II), Cu(II), Zn(II), Cd(II) and Hg(II)] salts are reported. The structural chemistry of these complexes is achieved via elemental analysis, spectral (UV, visible, and IR), thermal (DTA and TGA) as well as magnetic susceptibility. In these new octahedral complexes (Zn complex is tetrahedral), pyrazinamide acts as a bidentate ligand. Pyrazinamide complexes show higher activity than pyrazinamide for some strains. The geometry of the complexes is converted from Oh to Td during their thermal decomposition. The decomposition mechanisms are suggested and the thermodynamic parameters for the thermal decomposition steps are evaluated.

## 1 Introduction

**Figure 1:**
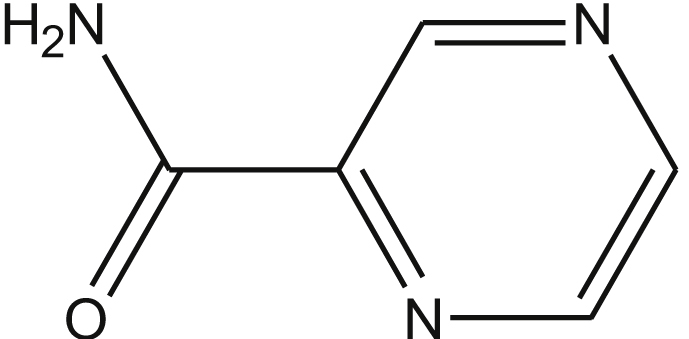
Structure of pyrazinamide, pyrazine-2-carboxamide.

Pyrazinamide has medicinal bacteriostatic and bactericidal effects on tuberculosis bacteria. The white crystalline drug pyrazinamide used to treat tuberculosis [1]. The systematic IUPAC name of pyrazinamide is pyrazine-2-carboxamide, Figure 1. The combination of pyrazinamide with other anti-tubercular drugs such as isoniazid [2] and rifampicin is highly effective and used in the treatment of mycobacterium tuberculosis. An antitubercular drug with Cu, Ag, Au, Zn, Hg, Fe and Co were synthesized and characterized by physicochemical and spectral methods. The ligand acts as a dianionic bidentate through oxygen and nitrogen centers [3]. Thermal analysis plays an important role in investigating the structure and the properties of metal complexes. The thermal transformations of pyrazinamide complexes with Cu and Cd can be described as a multi-step process consisting of crystalline phase transition, decomposition, melting and thermo-oxidation [4]. Masoud *et al.* reported the complexing properties and thermal behavior of some biologically active compounds [5, 6, 7, 8, 9, 10, 11, 12]. The main purpose of this work is to study the complexing properties and thermal behavior of pyrazinamide ligand and its metal complexes. Pyrazinamide can form a five-membered ring with metal ion during complexations which gives high stability to the formed complexes. The thermal decomposition mechanism is explained and the thermodynamic parameters are evaluated.

## 2 Experimental

**Figure 2:**
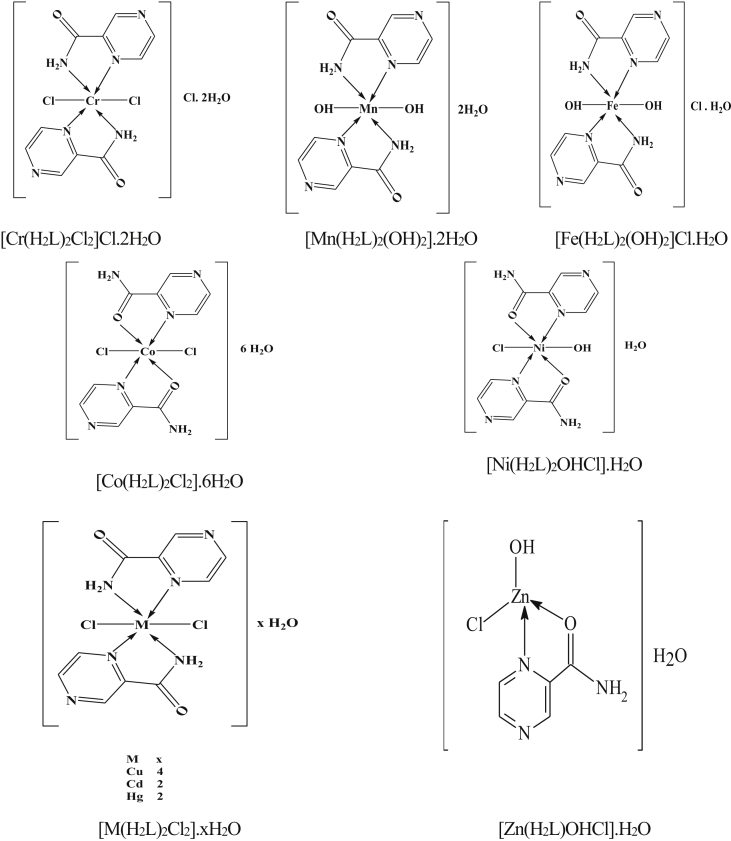
Proposed structures of pyrazinamide complexes.

Some metal [Cr(III), Mn(II), Fe(III), Co(II), Ni(II), Cu(II), Zn(II), Cd(II) and Hg(II)] chlorides are complexed with pyrazinamide ligand by a similar procedure. The metal chloride and ligand are dissolved in adequate volumes of ethanol separately. The molar amount of the metal chloride salt is mixed with the calculated amount of the ligand using different mole ratios (M:L) viz. 1:1 and 1:2. In each case, the reaction mixture is refluxed for about 5 min then left over-night, where the precipitated complexes were separated by filtration, then washed several times with a mixture of EtOH–H_2_O and dried in a vacuum desiccator over anhydrous CaCl_2_. The analytical results are given in Table 1. Elemental analyses of the synthesized complexes were done by the usual methods [13]. The metal contents were determined by using atomic absorption spectrophotometer (model 6650 Shimadzu) and complexmetrically with standard EDTA solution using the appropriate indicator as reported [14]. The chloride content of the complexes is determined by applying the familiar Volhard method [13]. The proposed structures of synthesized metal complexes were illustrated in Figure 2. The KBr disc IR spectra of the ligand and its complexes were measured over the frequency range 400–4000 cm^−1^ using Perkin-Elmer Spectrophotometer. The UV–Vis spectra of the solid complexes were measured in Nujol mull spectra [15]. Molar magnetic susceptibilities, corrected for diamagnetism using Pascal's constants were determined at room temperature (298 K) using Faraday's method. The instrument was calibrated with Hg[Co(SCN)_4_]. DTA and TGA analyses are carried out using a Shimadzu DTA/TGA-50. The rate of heating was 10 °C/min and the atmospheric nitrogen rate flow was 20 ml min^−1^. The biological screening of pyrazinamide and their metal complexes were examined against 5 microorganisms representing different microbial categories, {two Gram-positive (Staphylococcus Aureas ATCC6538P and Bacillus subtilis ATCC19659), two Gram negative (Escherischia coli ATCC8739 strain and Pseudomonas aeruginosa ATCC9027) and candida albicans as a fungi.

**Table 1:** Elemental analyses, m.pt, formula, stoichiometries and colors of pyrazinamide (H_2_L) complexes.

Complexes	Colour	Calculated/(Found)%				
		C	H	N	M	Cl
[Cr(H2L)2Cl2]Cl .2H2O	Pale green	27.24 (27.01)	3.18 (3.35)	19.07 (19.28)	11.80 (11.90)	24.18 (24.05)
[Mn(H_2_L)_2_(OH)_2_].2H_2_O	Beige	32.35 (32.60)	4.31 (4.02)	22.65 (22.43)	14.81 (14.61)	-
[Fe(H_2_L)_2_(OH)_2_]Cl.H_2_O	Dark orange	30.82 (31.00)	3.59 (3.39)	21.57 (21.28)	14.34 (14.66)	9.12 (9.32)
[Co(H_2_L)_2_Cl_2_].6H_2_O	Purple	24.79 (24.97)	4.55 (4.38)	17.36 (17.25)	12.18 (12.08)	14.67 (14.34)
[Ni(H_2_L)_2_OHCl].H_2_O	Pale blue	31.98 (31.52)	3.46 (3.71)	22.39 (22.18)	15.64 (15.58)	9.46 (9.25)
[Cu(H_2_L)_2_Cl_2_].4H_2_O	Olive green	26.52 (26.24)	3.98 (3.77)	18.56 (18.43)	14.04 (13.93)	15.69 (15.48)
[Zn(H_2_L)OHCl].H_2_O	White	23.18 (23.41)	3.09 (3.21)	16.22 (16.43)	25.25 (25.00)	13.71 (13.52)
[Cd(H_2_L)_2_Cl_2_ ].2H_2_O	White	25.78 (25.55)	3.01 (3.06)	18.05 (18.40)	24.15 (24.27)	15.26 (15.08)
[Hg(H_2_L)_2_Cl_2_].2H_2_O	White	21.68 (21.47)	2.53 (2.39)	15.17 (15.35)	36.23 (35.99)	12.83 (12.64)

All the complexes have m.pt. > 300 °C m.pt = melting point.

## 3 Results and discussion

### 3.1 IR spectra of pyrazinamide (H_2_L) and its metal complexes

There are two types of water molecules within the prepared complexes: coordinated and lattice water molecules. Coordinated water indicated that water molecules bonded to the metal through partially covalent bonds. Whereas the lattice water meant that water molecules were trapped in the crystalline lattice, either by weak hydrogen bonds to the anion or by weak ionic bonds to the metal, or by both. Generally, lattice water is absorbed at 3550-3200 cm^−1^ (asymmetric and symmetric OH stretching) [6]. From IR spectra of pyrazinamide and its complexes, one can notice that:

• The broad band at 3306-3380 cm^−1^ could be assigned to ν_O-H_ involved in hydrogen bond, due to the presence of lattice water molecules in outer sphere for all prepared complexes. For Mn, Fe, Ni and Zn complexes, these bands at 3540-3650 cm^−1^ could be taken as an indication of coordinated water molecules in the inner sphere and for OH-groups. This is proved by elemental and thermal analyses for these complexes. This is evident by ν_OH_, Table 2. However, coordinated water and OH-groups in these complexes are indicated by the appearance of metal-oxygen bands at 452-470 cm^−1^ region [16].

**Table 2:** Fundamental infrared bands (cm^−1^) of pyrazinamide (H_2_L) and their metal complexes.

Assignments	Pyrazinamide	CR- complex	MN- complex	FE- complex	CO- complex
Ν_O-H_ OF H_2_O	-	3306(b)	3564(b), 3320(b)	3650(b), 3380(b)	3370(B)
Ν_N-H_ STRETCHING	3413(vs), 3288(m)	3432(s), 3248(m)	3425(vs), 3257(m)	3426(s), 3268(m)	3407(S), 3285(W)
Ν_C=O_	1711(vs)	1701(9vs)	1709(vs)	1708(vs)	1695(VS)
Ν_C=N_	1609(s), 1579(m)	1586(s)	1609(m)	1609(m)	1590(M)
RING C–C STRETCHING	1523(w), 1475(m)	1522(w), 1473(m)	1522(w), 1475(m)	1523(w), 1475(m)	1522(W), 1474(W)
RING C–N STRETCHING	1436(s), 1376(9vs)	1423(w), 1369(vs)	1426(m), 1377(s)	1427(m), 1377(s)	1428(M), 1380(S)
Ν_C-N-C_ BENDING	870(s)	878(m)	870(m)	869(m)	869(M)
δ_N_–_H_	788(vs)	780(s)	788(s)	788(s)	787(S)
δ_C=O_	699(w)	696(w)	699(w)	698(w)	692(W)
Ν_C-C=N_ BENDING	522(vs)	538(s)	536(vs)	539(vs)	532(S)
ν_C_–_C_ STRETCHING	1053(w)	1052(w)	1054(w)	1055(w)	1059(W)
ν_M-O_	-	-	463(w)	470(w)	454(M)
ν_M-N_	-	428(s)	428(m)	420(s)	421(W)
ν_M-CL_	-	374(W)	**-**	**-**	389(W)
Assignments	NI- complex	CU- complex	ZN- complex	CD- complex	HG- complex
Ν_O-H_ OF H_2_O	3540(b), 3350(b)	3370(b)	3380(b), 3648(b)	3360(b)	3366(B)
Ν_N-H_ STRETCHING	3292(m)	3435(vs), 3270(w)	3406(vs), 3285(m)	3432(vs), 3269(w)	3425(S), 3267(M)
Ν_C=O_	1666(vs)	1706(vs)	1695(vs)	1701(vs)	1711(VS)
Ν_C=N_	1611(m), 1577(s)	1594(vs)	1590(s)	1587(s)	1612(M), 1574(W)
RING C–C STRETCHING	1536(m), 1498(w)	1519(m)	1524(w), 1473(w)	1518(w), 1473(w)	1520(W)
RING C–N STRETCHING	1425(m), 1401(m)	1429(vs), 1380(vs)	1429(s), 1381(vs)	1429(m), 1380(s)	1440(W), 1380(M)
Ν_C-N-C_ BENDING	866(s)	870(s)	871(s)	872(s)	869(S)
δ_N-H_	760(w)	796(vs)	787(m)	798(vs)	792(VS)
δ_C=O_	692(m)	708(m)	692(w)	705(w)	699(W)
Ν_C-C=N_ BENDING	538(m)	533(m)	533(s)	538(w)	538(S)
ν_C-C_ STRETCHING	1052(s)	1051(w)	1059(w)	1057(m)	1054(W)
ν_M-O_	459(m)	-	452(m)	-	-
ν_M-N_	420(w)	500(m)	432(w)	443(s)	426(S)
ν_M-CL_	372(W)	367(W)	391(W)	352(W)	360(W)

Abbreviations: vs (very strong), s (strong), m (medium), w (weak), b (broad).
• The carbonyl stretching vibrational band at 1711 cm^−1^ in the spectrum of pyrazinamide [17] have suffered from strong negative shift (1666-1699 cm^−1^) in Co, Ni and Zn complexes. This suggests that the coordination of the free ligand occurs through the oxygen atoms of carbonyl group. On the other hand, the band of δ_C=O_ appears at 699 cm^−1^ in the free ligand. In Co, Ni and Zn complexes, this band suffer from negative shift than the free ligand. This is proved that the oxygen atom of carbonyl group act as active site to make coordination in these complexes. In Cr, Mn, Fe, Cu, Cd and Hg complexes, ν_C=O_ have suffered only a slight negative shift (1701-1710 cm^−1^) on complex formation. This suggests that the carbonyl groups might be free of metal binding in these complexes.
• Pyrazinamide exhibits two broad bands at 3288 and 3413 cm^−1^ assigned to symmetric and asymmetric stretching vibrations of ν_NH_ group [18]. These bands still broad and overlapped with intramolecular hydrogen bonding in all prepared complexes due to the presence of water molecules. The symmetric and asymmetric ν_N-H_ bands are shifted either to lower or higher wave numbers in strong feature in all the prepared complexes except for Co, Ni and Zn complexes. The previous data indicated the participation of the –NH group in complexation to the metal ion in all complexes except for Co, Ni and Zn complexes.
• The ring C–N symmetric and asymmetric stretching vibration bands appear at 1376 and 1436 cm^−1^ while the deformation modes of ν_C-C=N_ bending band occurs at 522 cm^−1^. These bands are shifted on complexation. Also, the bands of ν_C=N_, ν_N-H_ and ν_C-N-C_ bending are affected on complexation.
• The presence of new bands in the region 420-500 cm^−1^ in the spectra of all the complexes (absent in pyrazinamide spectrum) assigned to ν_M-N_ These bands support the involvement of N-atom in coordination [19].The frequency in the range of 352–391 cm^−1^ has been observed in the case of all complexes except for Mn and Fe complexes which may be assigned to M-Cl band [6, 20] and this band is not present in pyrazinamide spectrum.
• Pyrazinamide can form chelate ring system due to this ligand have more than one point of attachment to the metal. This chelate ring system contains five membered ring including the metal ion through groups in the 1,4- position in pyrazinamide molecule [21]. These five membered rings give high stability to the new formed complexes. So, pyrazinamide acts as bidentate ligand through N atom of pyrazine ring and oxygen atom of amide group in case of Co, Ni and Zn complexes while through N atoms of pyrazine ring and amide group in all the rest complexes.

### 3.2 Electronic spectral and magnetic studies

The studied nujol mull electronic absorption spectral data (λ_max_, nm) at room temperature, effective magnetic moment values (μ_eff_, 298 K) and geometries of the studied complexes showed in Table 3. The nujol mull electronic absorption spectra for the pale green [Cr(H_2_L)_2_Cl_2_]Cl 2H_2_O complex, Table 3 showed three bands at 290, 310 and 600 nm due to ^4^A_2g_→^4^T_2g_ (F), ^4^A_2g_→^4^T_1g_(F) and ^4^A_2g_→^4^T_1g_(p) transitions, respectively. This complex has octahedral geometry in high spin state [22]. The complex has a magnetic moment value of 3.91 B.M. The proposed structure of this complex was justified depending on bidentate nature of pyrazinamide through N atoms of pyrazine ring and amide with the presence of two Cl ions in the inner sphere, two water molecules and one Cl ion in the outer sphere. The electronic absorption spectra for the beige manganese-complex, [Mn(H_2_L)_2_(OH)_2_] 2H_2_O, gave three bands at 290, 350 and 450 nm. The first peak is assigned to ^6^A_1g_→^4^A_1g_, while the second is due to ^6^A_1g_→^4^T_2g_ transition and the last band is due to ^6^A_1g_→^4^T_1g_ transition [23, 24]. This complex has the room temperature μ_eff._ value of 5.82 B.M. indicating octahedral geometry in high spin state d^5^ system with five unpaired electrons with sp^3^d^2^ hybridization. The structure of this complex is based on bidentate nature of pyrazinamide with the presence of two water molecules in the outer sphere. On the other hand the nujol mull electronic absorption spectra of the dark orange, [Fe(H_2_L)_2_(OH)_2_]Cl H_2_O complex, Table 3, showed four bands at 270 (m), 344 (m), 412 (m) and 472 (b) nm. These bands are due to CT (t_2g_ → π*) and CT (π → e_g_). Its room temperature μ_eff_ value of 5.90 B.M typified the existence of octahedral geometry in high spin state with sp^3^d^2^ hybridization [4]. The structure of this complex is based on bidentate nature of pyrazinamide with the presence of one water molecule and one chloride ion in the outer sphere. The purple [Co(H_2_L)_2_Cl_2_] 6H_2_O complex, Table 3, gave bands at 250, 300 and 392 and 478 nm. The first two bands are of metal to ligand charge transfer nature and the latter broad band is assigned to ^4^T_1g_(F) → ^4^T_1g_(P) transition typified *O*_*h*_ geometry [5]. The magnetic moment value is 3.91 B.M. indicated high spin nature of the complex with three unpaired electrons. The proposed structure of this complex depended on bidentate nature of the organic molecule with the presence of two Cl ions in the inner sphere and six water molecules in the outer sphere. The nujol mull electronic spectra of the pale blue [Ni(H_2_L)_2_OHCl]H_2_O complex, Table 3, gave four bands at 270, 340, 405 and 610 nm. The latter broad band is taken as diagnostic for *O*_*h*_ symmetry and assignable to ^3^A_2g_(F)→^3^T_1g_(F) and ^3^A_2g_(F)→^3^T_1g_(P). The broadness is attributed to the existence of more than d-d transition overlapped with each other [7]. The room temperature magnetic moment value for this complex is 2.81 B.M to assign high spin octahedral configuration with the presence of two unpaired electrons. The structure of this complex is based on bidentate nature of the organic compound, one Cl ion in the inner sphere and one water molecule in the outer sphere. The electronic spectral data of the olive green [Cu(H_2_L)_2_Cl_2_] 4H_2_O complex, showed bands at 270, 350, 420 and 700 nm. The latter broad band is assigned to the transition ^2^E_g_ → ^2^T_2g_ (D) transition assignable to octahedral environment, Table 3. The room temperature magnetic moment value for this complex is 1.73 B.M typified the existence of octahedral geometry. The proposed structure depends on the bidentate nature of the organic compound with the presence of two Cl ions in the inner sphere and two water molecules in the outer sphere.

**Table 3:** Nujol mull electronic absorption spectra (nm), room temperature magnetic moment values (μ _eff_,298 K) B.M and geometries of complexes.

COMPLEX	Λ _MAX_ (NM)	Μ _EFF_	GEOMETRY
[CR(H_2_L)_2_CL_2_]CL 2H_2_O	250, 310, 600	3.91	*Oh*
[MN(H_2_L)_2_(OH)_2_] 2H_2_O	250, 350, 455	5.82	*Oh*
[FE(H_2_L)_2_(OH)_2_]CL H_2_O	270, 344, 412, 472	5.90	*Oh*
[CO(H_2_L)_2_CL_2_] 6H_2_O	250, 300, 392, 478	3.60	*Oh*
[NI(H_2_L)_2_OHCL]H_2_O	270, 340, 405, 610	2.80	*Oh*
[CU(H_2_L)_2_CL_2_] 4H_2_O	270, 350, 420, 700	1.73	*Oh*
[ZN(H_2_L)OHCL] H_2_O	**-**	zero	*Td*
[CD(H_2_L)_2_CL_2_ ]2H_2_O	**-**	zero	*Oh*
[HG(H_2_L)_2_CL_2_] 2H_2_O	**-**	zero	*Oh*

### 3.3 Thermal analysis investigations

**Scheme 1:**
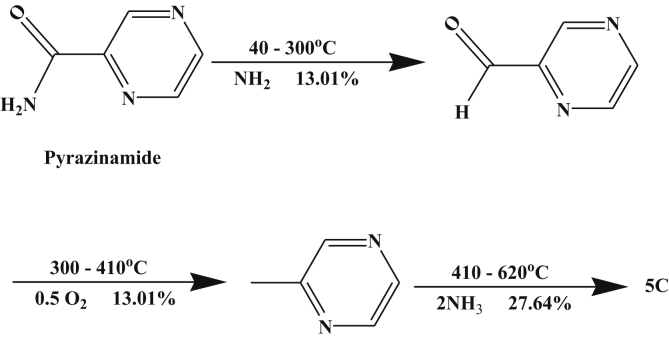
Thermolysis of pyrazinamide.

From TGA of pyrazinamide (H_2_L) and its complexes, Table 4, pyrazinamide decomposition occurs in three steps until 620 °C end with formation of carbon residue as a final product. The decomposition equation may be supposed as in scheme 1. The DTA curve, pyrazinamide decomposition occurs in four steps. First step is endothermic at 643K and the last three are exothermic at 373, 438 and 726K with activation energies 27.72, 52.54, 762.79 and 510.27 kJ/mol with orders 0.79, 1.38, 1.37 and 1.06, respectively, indicating the first order type of these steps. The TGA thermogram has one peak in temperature range 40–300 °C which corresponds to elimination of the NH_2_ group.

**Table 4:** DTA analysis of pyrazinamide and their metal complexes.

Compound	Type	Tm (°K)	E_a_ kJ mol^−1^	n	α_m_	ΔS^#^ kJ K^−1^ mol^−1^	ΔH^#^ kJ mol^−1^	10^3^ Z S^−1^	Temp. (°C) TGA	Wt. Loss %		Assignment
										Calc	Found	
Pyrazinamide	Exo	373	27.72	0.79	0.67	-0.305	-113.81	0.009	40–300	13.01	12.98	Elimination of NH_2_
	Exo	438	52.54	1.38	0.57	-0.302	-132.49	0.014				
	Endo	643	762.79	1.37	0.57	-0.287	-184.31	0.142	300–410	13.01	13.23	Loss of 0.5 O_2_
	Exo	726	510.27	1.06	0.62	-0.292	-211.99	0.085	410–620	27.64	28.03	Loss of 2NH_3_ and formation of carbon residue.
Cr-complex	Exo	393	14.55	0.70	0.69	-0.303	-119.36	0.011	30–120	8.17	8.11	Dehydration of 2H_2_O
	Endo	453	76.49	1.47	0.56	-0.302	-136.91	0.015	120–260	36.89	36.88	Removal of 3HCl, CH_3_NH_2_ and 0.5N_2_O.
	Exo	783	192.05	1.15	0.60	-0.302	-236.66	0.027	260–600	12.71	12.14	Loss of 2N_2_ and formation of 0.5 Cr_2_O_3_ +9C.
Mn- complex	Exo	343	35.89	0.73	0.69	-0.302	-103.45	0.013	30–100	4.85	5.36	Dehydration of H_2_O
	Endo	407	9.09	1.26	0.59	-0.316	-128.56	0.003	100–135	14.56	15.56	Dehydration of 3H_2_O
	Exo	473	13.77	1.25	0.58	-0.315	-148.95	0.004	135–220	11.86	11.66	Loss of N_2_O
	Endo	517	144.39	1.71	0.55	-0.297	-153.47	0.034	220–300	14.70	14.51	Removal of 2NH_3_ and N_2_ with formation of MnO+ 10C
Fe- complex	Exo	323	25.86	1.63	0.54	-0.303	-97.97	0.009	40–210	25.57	25.12	Dehydration of 3H_2_O and loss of HCl.
	Exo	403	52.54	1.62	0.54	-0.301	-121.35	0.016	210–260	20.34	20.64	Removal of N_2_O and C_2_H_4_.
	Endo	498	196.71	0.94	0.64	-0.294	-146.24	0.048	260–600	47.46	42.95	Loss of 2N_2_ and 8C with formation of FeO
Co-complex	Exo	393	11.88	1.05	0.62	-0.313	-123.03	0.004	50–180	22.36	22.11	Dehydration of 6H_2_O
	Endo	511	46.19	1.06	0.62	-0.306	-156.43	0.011	180–600	54.29	54.23	Loss of 2HCl, 2NH_3_, N_2_, N_2_O and 2C_2_H_11_ with formation of CoO
	Endo	733	109.69	1.05	0.62	-0.305	-223.52	0.018				
Ni- complex	Exo	365	26.91	0.81	0.67	-0.305	-111.34	0.009	30–180	19.33	19.56	Dehydration of 2H_2_O and loss of HCl.
	Endo	423	56.84	1.44	0.56	-0.301	-127.44	0.016				
	Exo	563	20.11	1.81	0.55	-0.315	-177.15	0.004	180–400	16.53	16.31	Removal of 2NH_3_ and N_2_.
	Exo	773	97.07	1.26	0.59	-0.307	-237.19	0.015	400–600	11.73	11.56	Elimination of N_2_O and formation of NiO +10C
Cu- complex	Exo	335	28.19	0.94	0.64	-0.303	-101.58	0.011	38–120	15.90	15.77	Dehydration of 4H_2_O
	Endo	393	61.39	1.15	0.61	-0.299	-117.66	0.019	120–210	16.13	16.01	Loss of HCl.
	Exo	503	37.12	1.85	0.52	-0.308	-154.77	0.009	210–370	9.72	9.51	Removal of N_2_O
	Exo	653	38.87	2.77	0.44	-0.312	-203.50	0.007	370–600	13.26	13.01	Elimination of 2NH_3_ and N_2_ with formation of CuO+ 10C

**Figure 3:**
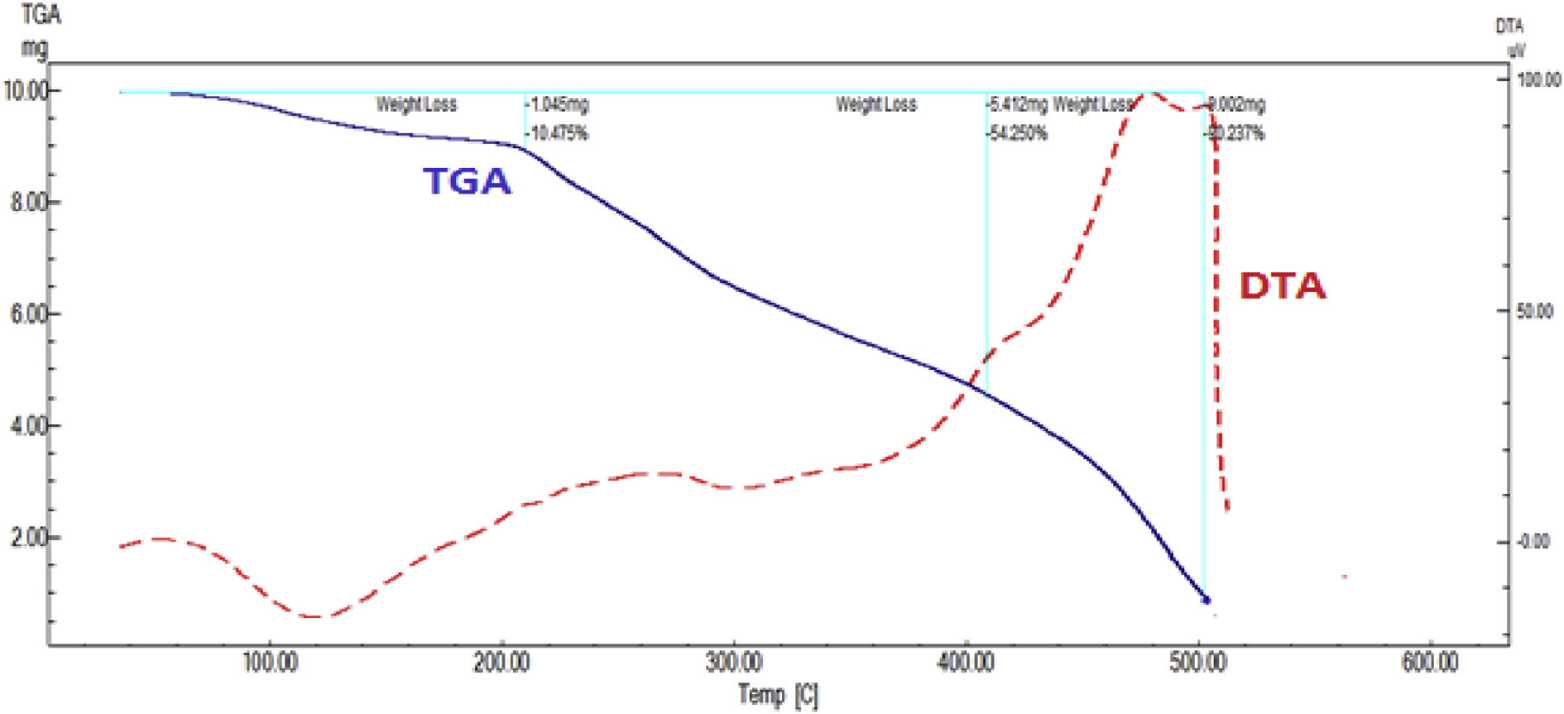
TGA and DTA OF Cr –complex.

The DTA data of Cr-complex, Figure 3 and Table 4, showed three peaks at 393, 453 and 783 K with activation energies 14.55, 76.49 and 192.05 kJ/mol, respectively. The orders of reactions are 0.70, 1.47 and 1.15 indicating 1^st^ order.

**Scheme 2:**
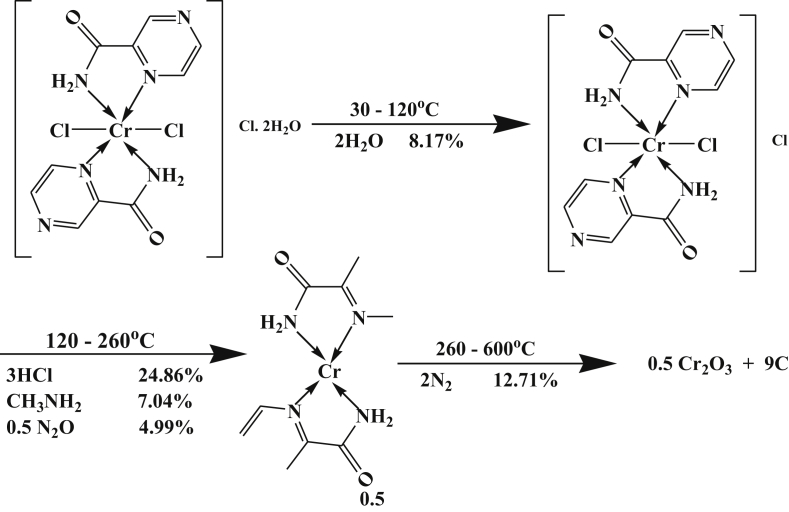
Thermolysis of Cr-pyrazinamide complex.

All peaks are exothermic except the second one is endothermic in nature. The TGA data confirmed these results where it also gives three peaks. The first one is due to dehydration of two lattice water molecules while the second one is due to elimination of 3HCl, CH_3_NH_2_ and 0.5N_2_O. The last step corresponds to elimination of 2N_2_ and formation of 0.5 Cr_2_O_3_ +9C as a final product with percent 41.77 (41.98). The mechanism of decomposition is represented in the following scheme 2.

On the other hand, the DTA data of Mn-complex, Table 4, showed four peaks, at 343, 407, 473 and 517 K with activation energies 35.89, 9.09, 13.77 and 144.39 kJ/mol, respectively. The orders of reactions are 0.73, 1.26, 1.25 and 1.71, respectively. All peaks are of the first order type except the last one is second order. The first and third peaks are of exothermic type while the second and the last peaks are of endothermic agitation types [25].

**Scheme 3:**
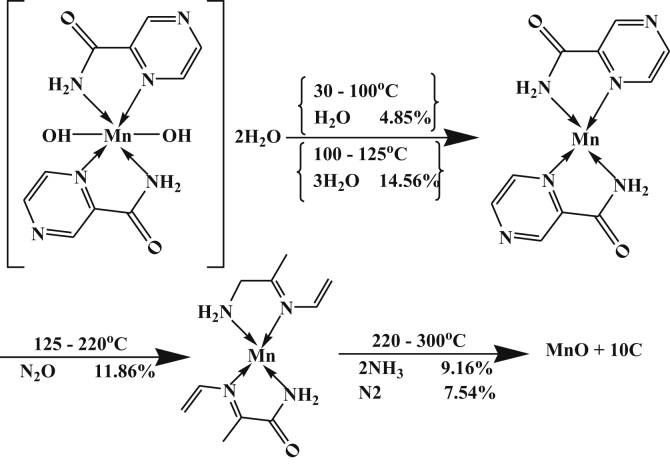
Thermolysis of Mn-pyrazinamide complex.

This can be proved by TGA data, which gave four well-defined peaks; the first two's are due to the evolved of lattice and coordinated water molecules. The last two's are due to the decomposition steps and formation of MnO +10C as a final product. The mechanism of decomposition is represented in scheme 3.

**Scheme 4:**
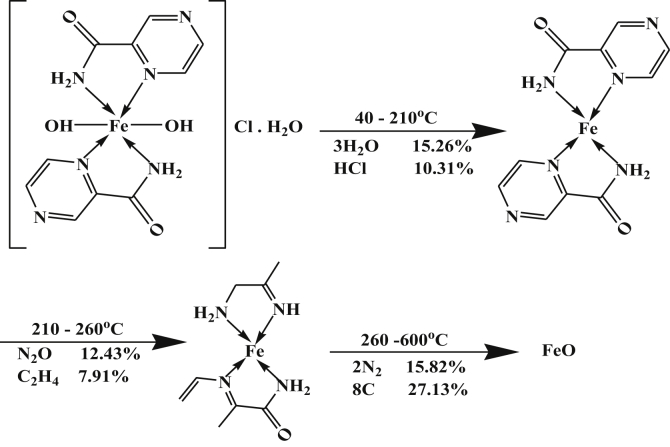
Thermolysis of Fe-pyrazinamide complex.

The DTA data of Fe-complex, Table 4, showed three peaks, at 323, 403 and 498K with activation energies 25.86, 52.54 and 196.71 kJ/mol, respectively. The orders of reactions are 1.63, 1.62 and 0.94. All peaks of second order reactions except for the last one is of the first order type. All peaks are of exothermic type except the last one is of endothermic type. This can be proved by TGA data that gave three well-defined peaks, the first one corresponds to dehydration of outer and inner water molecules and loss of HCl. The last two's are due to the decomposition steps and formation of FeO as a final product. The mechanism of decomposition is represented in scheme 4.

The Co-complex, Table 4, showed three peaks at 393, 511 and 733 K with activation energies of 11.88, 46.19 and 109.69 kJ/mol, their calculated reaction orders are 1.05, 1.06 and 1.05 indicating first order. All peaks are endothermic except the first one is exothermic. However, the TGA data gave two peaks; the first one is due to dehydration process of lattice water molecules while the last peak is due to the decomposition step ended with the formation of CoO as a final product.

**Scheme 5:**
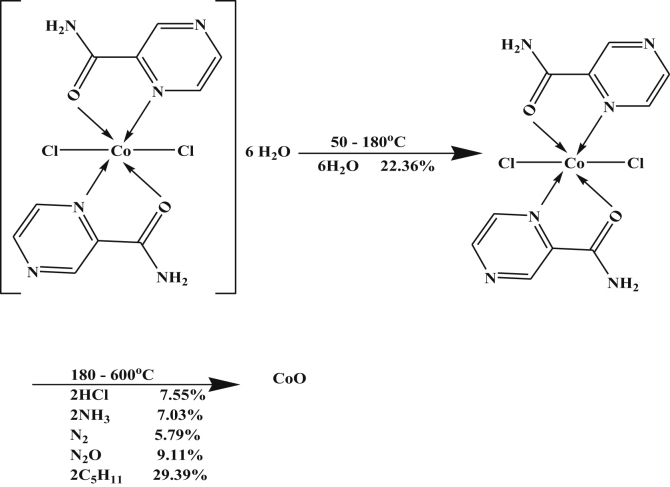
Thermolysis of Co-pyrazinamide complex.

The two DTA endothermic peaks in the temperature range 180–600 °C overlapped in the TGA to give one peak that corresponds to elimination of small molecules with formation of CoO as a final product. The mechanism of decomposition is represented as in scheme 5.

**Scheme 6:**
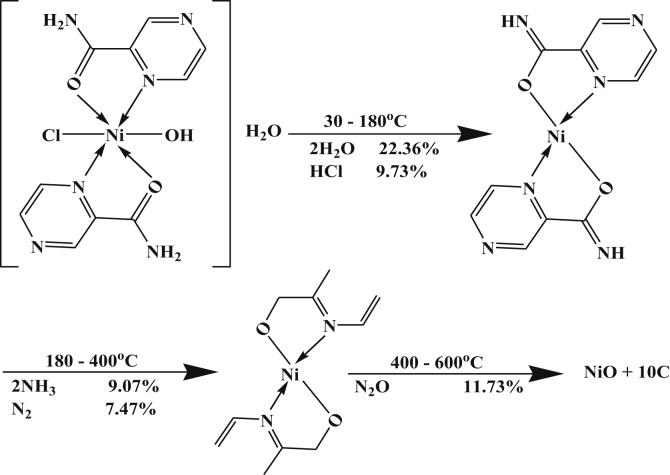
Thermolysis of Ni-pyrazinamide complex.

The DTA data of Ni-complex, Table 4, gave four peaks. Three of them are exothermic at 365, 563 and 773 K with activation energies of 26.91, 20.11 and 97.07 kJ/mol. The last peak is endothermic in nature at 423 K with activation energy of 56.84 kJ/mol. All the data typified first order reactions except the third exothermic peak is of the second order type. The TGA data gave three peaks, the first two peaks from DTA in the temperature range 30–180 °C overlapped to give one peak in TGA which corresponds to dehydration process of water molecules [26] and loss of HCl. The last three strong peaks are due to decomposition steps with the formation of NiO +10C as a final product. The mechanism of decomposition is summarized in scheme 6.

However, the DTA thermogram Cu-complex, Table 4, showed four well defined peaks at 335, 393, 503 and 653 K from the DTA data with activation energies of 28.19, 61.39, 37.12 and 38.87 kJ/mol. All peaks are exothermic except the second one is endothermic.

**Scheme 7:**
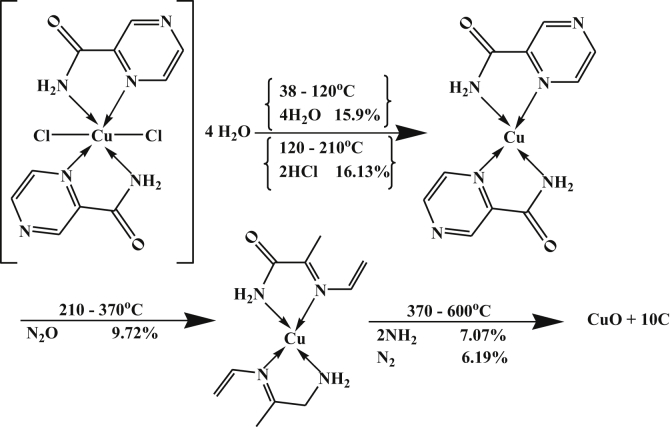
Thermolysis of Cu-pyrazinamide. complex.

Their orders of reactions are 0.94, 1.15 and 1.05 (indicating 1^st^ order), 1.85 (indicating 2^nd^ order) and 2.77 (indicating third order), respectively. Also, the TGA data gave four peaks, The first one is due to a dehydration reaction of lattice water molecules and the last three strong peaks are due to the decomposition reactions [27, 28] ended with the formation of CuO +10C as a final product with percent 44.09 (44.45). The mechanism of decomposition is summarized in scheme 7.

**Figure 4:**
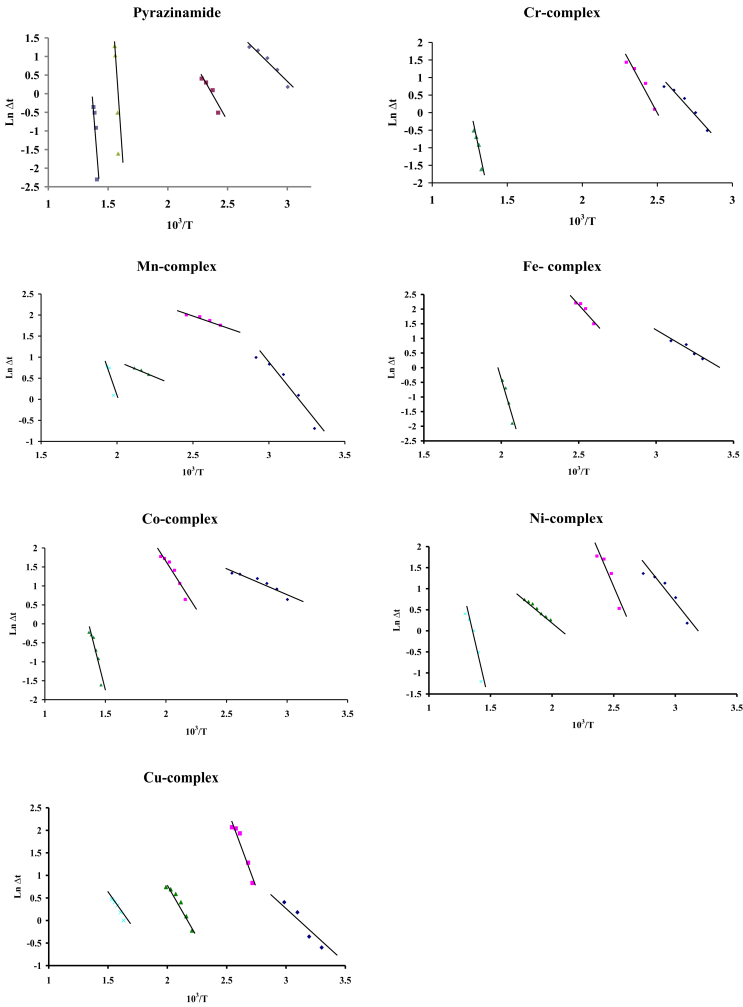
ln Δt against 10^3^/T relation of pyrazinamide and its complexes.

The change of entropy, ΔS^#^, values for all complexes is nearly of the same magnitude and lies within the range (-0.287 to -0.316) kJ K^−1^ mol^−1^.So, the transition states are more ordered, i.e. in a less random molecular configuration, than the reacting complex. The thermal reaction calculations showed the remaining fraction, n, also confirming that the reactions proceeded in complicated mechanisms. The calculated values of the collision number, z showed a direct relation to E_a_ as illustrated in Table 4. Based on least square calculations, the ln ΔT versus 10^3^/T plots for all complexes, Figure 4, gave straight lines from which the activation energies were calculated according to the methods of Piloyan et al. [29]. The order of chemical reactions (n) was calculated via the peak symmetry method [30].

**Figure 5:**
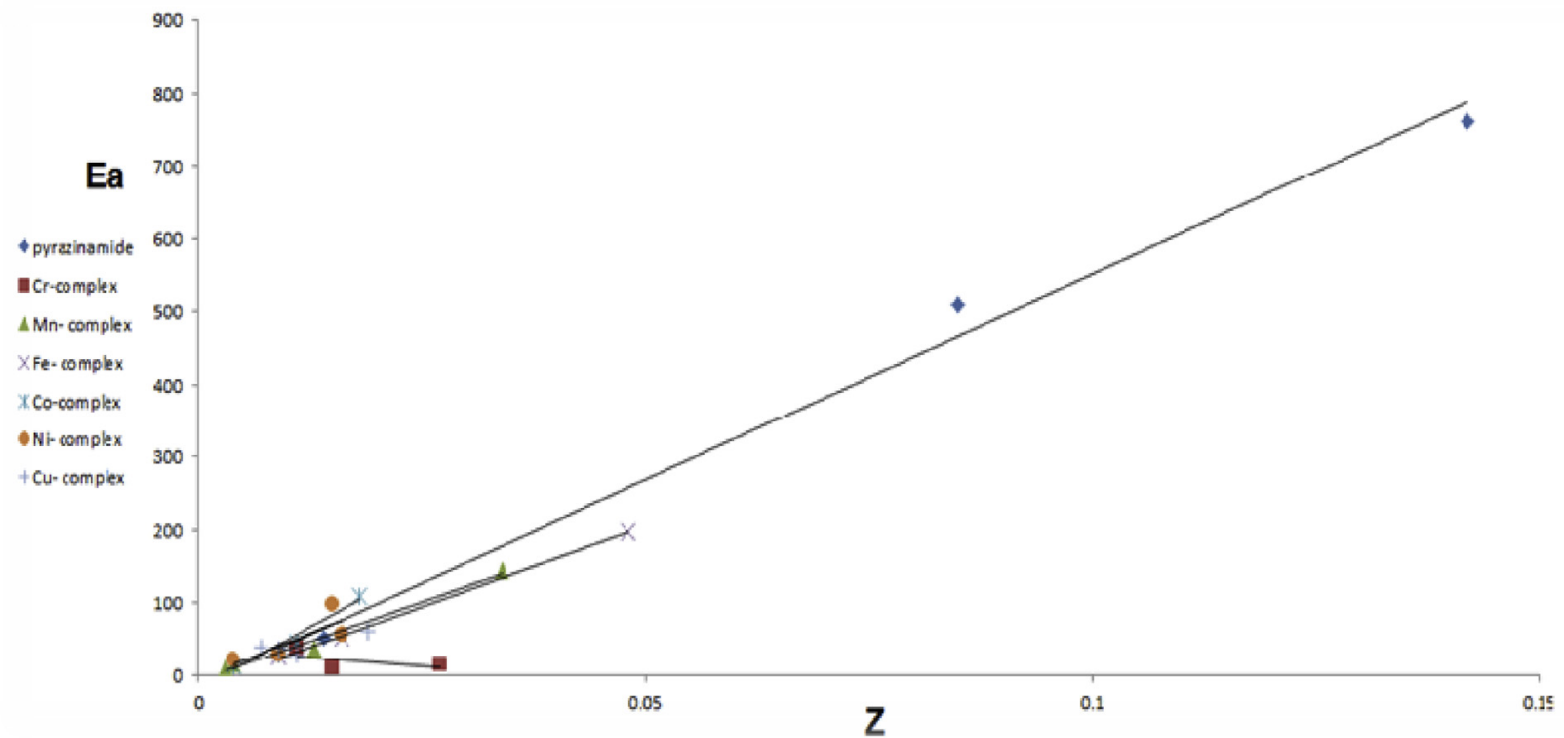
Relation between Z and E_a_ for pyrazinamide and their complexes.

The activation energies E_a_ of the thermal decomposition steps, for pyrazinamide and its complexes represent collision number Z relationship, Figure 5. There is a direct relationship. When some suitable particles of the reactant hit each other, only significant chemical change obtained by a certain percentage of collisions. The successful collisions have enough energy, known as E_a_ at the moment of impact to break the preexisting bonds and form all new bonds. This results in the products of the reaction. The rate of reactions affected by increasing the concentration of the reactant particles or raising the reaction temperature. This cause more collisions and therefore many successful collisions cause raise in reaction rate.

**Figure 6:**
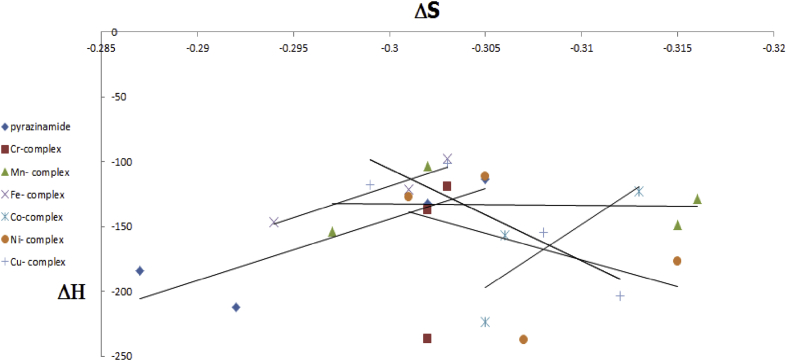
Relation between Δ*H* and Δ*S* for pyrazinamide and their complexes.

The relationship between Enthalpy (Δ*H*) and entropy (Δ*S*), Figure 6 is random and depend on each reaction. Enthalpy (Δ*H*) is a measure of amount of released or absorbed energy during a chemical reaction. Energy, in the form of heat, is released in an exothermic reaction, and the change in enthalpy is negative, *-H*. On the other hand, energy, in the form of heat, is absorbed in an endothermic reaction, and this time the change in enthalpy is positive, *+H*. Entropy (Δ*S*) is a measure of disorder or randomness in the system. In nature, a messy room is far more favored than a neat, ordered room, and when disorder increases, we have *+ S*.

## 4 Biological activity

From the following observations, Table 5 it's indicated that all the investigated compounds have higher positive antibacterial activity compared to antifungal activity. [CU(H2L)2Cl2].4H2O showed higher activitiy for Pseudomonas aeruginosa, Candida albicans, Escherichia coli, Staphylococcus aureus and Bacillus subtilis. It revealed by the diameter of its inhibition zone, [Zn(H2L)OHCl].H2O complex showed higher activity to Escherichia coli and Bacillus subtilis. It showed activity in the same range of ceftazidime for Candida albicans and Pseudomonas aeruginosa. Most of the metal complexes have higher activity than the free ligands such increased activity of the metal chelates could be explained on the bases of overtoneۥ s concept and chelation theory [31]. The cell permeability the lipidmembrane that surrounds the cell favours the passage of only lipid soluble materials on the basis that liposolubility is an important factor that controls antimicrobial activity.

**Table 5:** The antifungal activity of the free pyrazinamide and its complexes against some reference strains expressed in absolute activity (AU).

Compounds	Blank	Candida albicans	Escherischia coli	Pseudomonas aeruginosa	Staphyllococcus aureus	Bacillus subtilis
[CU(H_2_L)_2_Cl_2_].4H_2_O	8	12	16	12	14	15
[Zn(H_2_L)OHCl].H_2_O	8	8	14	8	14	15
pyrazinamide	8	8	12	8	12	11
Ciprofloxacin	9	30	30	30	30	17^a^

a Clotrimazole is the reference for bacillus subtilis.

## 5 Conclusion

Pyrazinamide reacts with Cr(III), Mn(II), Fe(III), Co(II), Ni(II), Cu(II), Zn(II), Cd(II) and Hg(II) ions to form complexes by acting as a bidentate ligand and all complexes were of octahedral geometry except Zn complex that is of tetrahedral. The thermal decomposition steps of the most complexes ended with the formation of metal oxides and carbon residue as a final product. The geometries of complexes may be converted from *O*_*h*_ to *T*_*d*_ during the thermal decomposition. The first steps always were corresponding to dehydration process of lattice and coordinated water molecules followed by ligand decomposition steps. Decomposition mechanisms were suggested.

## Declarations

### Author contribution statement

Alaa E. Ali: Conceived and designed the experiments.

Sherif A. Kolkalia: Performed the experiments; Wrote the paper.

Gehan S. Elassala: Analyzed and interpreted the data.

Esam A. Mohamed: Contributed reagents, materials, analysis tools or data.

### Funding statement

This research did not receive any specific grant from funding agencies in the public, commercial, or not-for-profit sectors.

### Competing interest statement

The authors declare no conflict of interest.

### Additional information

No additional information is available for this paper.
